# Preparative Isolation of Seven Diterpenoid Alkaloids from *Aconitum coreanum* by pH-Zone-Refining Counter-Current Chromatography

**DOI:** 10.3390/molecules190812619

**Published:** 2014-08-19

**Authors:** Xueyong Wang, Xikai Shu, Xiao Wang, Jinqian Yu, Feng Jing

**Affiliations:** 1 College of Chinese Mareria Medica, Beijing University of Chinese Medicine, Beijing 100102, China; E-Mail: wxyph.d@163.com; 2 Shandong Analysis and Test Center, Shandong Academy of Sciences, 19 Keyuan Street, Jinan 250014, China; E-Mails: shuxikai5@163.com (X.S.); yujinqian87528@126.com (J.Y.); gaoaixiangjf@163.com (F.J.)

**Keywords:** alkaloids, *Aconitum coreanum*, pH-zone-refining counter-current chromatography

## Abstract

The aim of this paper was to seek an efficient method to preparative separation of alkaloid compounds from *Aconitum coreanum* (Guanbaifu), a well-known traditional Chinese medicinal plant for heart disease. Seven alkaloid compounds were successfully purified by pH-zone-refining counter-current chromatography with two-phase solvent system of petroleum ether–ethyl acetate–methanol–water (5:5:1:9, v/v/v/v), 10 mM triethylamine in upper phase and 10 mM hydrochloric acid in lower phase. From 3.5 g of crude extract, 356 mg of Guanfu base I, 578 mg of Guanfu base A, 74 mg of atisine, 94 mg of Guanfu base F, 423 mg of Guanfu base G, 67 mg of Guanfu base R and 154 mg of Guanfu base P were obtained with the purity of 96.40%, 97.2%, 97.5%, 98.1%, 98.9%, 98.3% and 98.4%. Their chemical structures were identified by TOF-MS and ^1^H-NMR. This study indicated that pH-zone-refining counter-current chromatography was an efficient method for separating the kind of alkaloids with low absorbance values.

## 1. Introduction

*Aconitum coreanum* (Lèvl) Rapaics (Guanbaifu in Chinese) grows in Primorskii Krai, south China, and Korea [1]. This herb has been widely used to treat various kinds of diseases such as cardialgia, facial distortion, epilepsy, migraine headache, tetanus, infantile convulsion and rheumatic arthralgia [2]. The major active constituents of *Aconitum coreanum* are considered to be Guanfu base A (GFA), Guanfu base I (GFI) and Guanfu base G (GFG) [3,4]. The early research showed that GFA could effectively terminate the fibrillation induced by bilateral vagus nerve stimulation with the total effective rate of 87.5% [5], it has strong antiarrhythmic effect and can effectively bind atrial fibrillation [5,6]. It has been developed into a neo-type antiarrhythmic drug.

**Figure 1:**
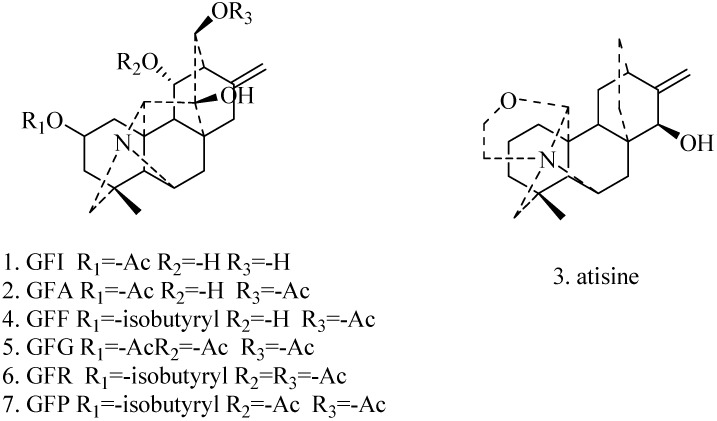
Chemical structures of compounds from *Aconitum coreanum* (Lèvl.) Rapaics.

The alkaloids of *Aconitum coreanum* are difficult to separate, as the structures and physico-chemical properties are similar and its absorbance value is very low (about 200 nm). However, pH-zone-refining counter-current chromatography (pH-zone-refining CCC) which was a liquid-liquid chromatography introduced by Ito [7,8] is a very good solution to this problem, as the main alkaloids can be separated by little peaks which are formed by minor impurities. So, pH-zone-refining CCC is an excellent technique for separating this type of alkaloids. The classical methods such as column chromatography and high-performance liquid chromatography (HPLC) are usually time consuming, requiring multiple chromatography steps. The method of pH-zone-refining CCC has been used to isolate bioactive compounds in many natural products [9,10,11] and has many important advantages including larger sample loading capacity, shorter separation time, high concentration of fractions and concentration of minor impurities compared to conventional counter-current chromatography method [7]. This paper aims to report the advantages of these technologies, namely pH-zone-refining CCC for separation the seven main alkaloids (Figure 1) from *Aconitum coreanum* (Lèvl.) Rapaics. The critical parameter of the solvent system of pH-zone-refining CCC was optimized.

## 2. Results and Discussion

### 2.1. Selection of Two-Phase Solvent System

The choice of a suitable two-phase solvent system is the first and critical step in a pH-zone refining CCC experiment. According to the rules introduced by Lei Fang [12], the suitable solvent system for a successful pH-zone refining CCC of an alkaloid mixture should provide ideal partition coefficient (*K*) values in acidic (*K*_acid_ << 1) and *K* > 1 as well as good solubility of the sample in the solvent system.

The Pet–EtOAc–MeOH–H_2_O is one of the main two-phase solvent systems used for pH-zone refining CCC separation [8,12]. Moreover, Pet–EtOAc–MeOH–H_2_O could provide a broad polarity range and a good solubility of the sample. Thus, in our research, several Pet–EtOAc–MeOH–H_2_O solvent systems at different solvent ratios were screened using the new method, and the values of *K* and *K*_acid_ for the seven alkaloids in different solvent systems were summarized in Table 1. The results indicated that the two-phase solvent system composed of Pet–EtOAc–MeOH–H_2_O with the volume ratios of 5:5:2:8 and 5:5:1:9 provided ideal *K* and *K*_acid_ values, which could be suitable for the separation.

**Table 1:** The partition coefficient (*K*) values in both acidic and alkaline of different systems.

Solvent System (Pet–EtOAc–MeOH–H_2_O) (v/v/v/v)		*K*_1_	*K*_2_	*K*_3_	*K*_4_	*K*_5_	*K*_6_	*K*_7_
5:5:4:6	*K*	0.75	0.82	1.05	1.01	1.34	1.38	1.55
	*K*_acid_	0.07	0.05	0.08	0.07	0.08	0.09	0.11
5:5:2:8	*K*	1.52	1.52	1.75	1.78	2.12	2.23	2.25
	*K*_acid_	0.12	0.12	0.14	0.12	0.15	0.16	0.19
5:5:1:9	*K*	1.65	1.69	1.87	1.91	2.21	2.34	2.39
	*K*_acid_	0.15	0.16	0.18	0.18	0.21	0.23	0.25

**Figure 2:**
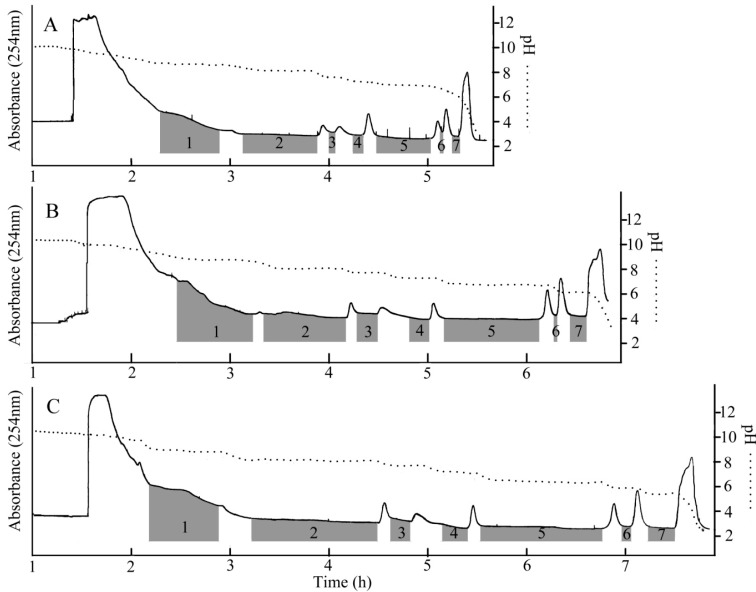
pH-zone-refining CCC chromatograms of preparative separation of alkaloids compounds from *Aconitum coreanum* (Lèvl.) Rapaics; Condition: (**A**) Two-phase solvent system: Pet–EtAc–MeOH–H_2_O (5:5:4:6, v/v/v/v); the retention of the stationary phase: 53%. (**B**) Two-phase solvent system: Pet–EtAc–MeOH–H_2_O (5:5:3:7, v/v/v/v); the retention of the stationary phase: 55%. (**C**) Two-phase solvent system: Pet–EtAc–MeOH–H_2_O (5:5:1:9, v/v/v/v); the retention of the stationary phase: 62%. The same condition: 10 mM TEA in stationary phase and 10 mM HCl in lower phase; flow rate: 2 mL/min; detection wavelength: 254 nm; revolution speed: 850 rpm; sample size: 3.5 g.

After three two-phase solvent systems were tested, the results were shown in Figure 1 which indicated that the target compounds were separated step by step. When the two-phase solvent system Pet–EtOAc–MeOH–H_2_O (5:5:4:6, v/v/v/v) (10 mM TEA in upper phase and 10 mM HCl in lower phase) was used, as shown in Figure 2A, the target compounds were eluted quickly that compounds **1**, **2** and **6** did not separate well. Compounds **1** and **2** were only partly separated and the purity of compound **6** was lower than 80% as determined by HPLC.

So, Pet–EtOAc–MeOH–H_2_O (5:5:2:8, v/v/v/v) (10 mM TEA in upper phase and 10 mM HCl in lower phase) was selected to test. As shown in Figure 2B, compounds **1** and **2** were separated well. However, the purity of compounds **1** and **6** was only about 89% (HPLC analysis chromatograms were not shown). To enhance the purity of compounds **1** and **6**, the eluting speed should be reduced.

**Figure 3:**
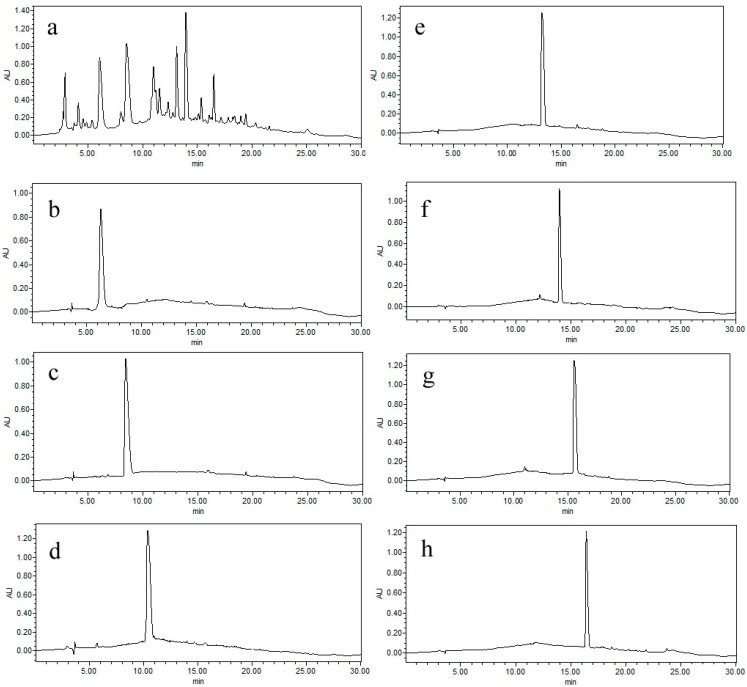
HPLC chromatograms: (**a**) Crude extracts from *Aconitum coreanum*; (**b**) GFI; (**c**) GFA; (**d**) atisine; (**e**) GFF; (**f**) GFG; (**g**) GFR; (**h**) GFP; Column: Warters SymmetryShield^TM^ RP18 column (250 mm × 4.6 mm I.D., 5 μm); Mobile phase: Acetonitrile and 2 mg/mL sodium 1-heptansulfonate (including 0.2% triethylamine and the pH was adjusted to 3.0 with phosphoric acid) was used as the mobile phase in gradient elution mode as follows: Acetonitrile: 0–10 min, 10 to 30; 10–20 min, 30–60; 20–21 min, 60 to 10; 21–30 min, 10; flow rate: 1.0 mL/min. Detection wavelength: 200 nm.

So the ratio of methanol was reduced and the solvent system of Pet–EtOAc–MeOH–H_2_O (5:5:1:9, v/v/v/v) (10 mM TEA in upper phase and 10 mM HCl in lower phase) was selected to test. The compounds **1**–**7** were separated well as Figure 2C showed and the purity was all over 95%. A typical chromatogram of pH-zone-refining CCC was obtained. The pH measurement also revealed flat pH zones which correspond to the above absorbance plateaus and suggested successful separations of the components. Exactly 356 mg of GFI (**1**), 578 mg of GFA (**2**), 74 mg of atisine (**3**), 94 mg of GFF (**4**), 423 mg of GFG (**5**), 67 mg of GFR (**6**), and 154 mg of GFP (**7**) were obtained from 3.5 g crude extract, with the purity of the purity of 96.40%, 97.2%, 97.5%, 98.1%, 98.9%, 98.3% and 98.4% as determined by HPLC as showed in Figure 3.

Given the observed elution results in Figure 2, reducing the amount of bridge solvent (methanol) could be successfully applied to pH-zone refining CCC. As the bridge solvent (methanol) was reduced, the resolution and time of separation increased. The results indicated that the compounds could be successfully separated by adjusting the bridge solvent (methanol).

In the past, the alkaloids from *Aconitum coreanum* have been separated by the conventional high-speed CCC method [13]. The present system utilizing pH-zone-refining CCC could separate the target compounds at much higher purity and larger amounts in a single run, indicating the powerful separation capability of pH-zone-refining CCC. The compartion of two separation methods is shown in Table 2, which demonstrated that pH-zone-refining CCC had many advantages over conventional CCC. It had an over 10-fold increase in sample-loading capacity, high purity, high yield, high recovery and high concentration of the collected fractions.

**Table 2:** The compartion of two separation methods.

Results	Standard HSCCC						pH-Zone-Refining CCC						
	GFI	GFA	atisine	GFF	GFG	GFP	GFI	GFA	atisine	GFF	GFG	GFR	GFP
Purity (%)	95.5	95.8	98.9	91.5	95.7	96.9	97.1	98.5	97.4	98.7	99.1	98.1	98.7
Yield (mg)	25.7	11.9	8.9	9.5	9.2	10.4	356	578	74	94	423	67	154
Sample Size	2 g						3.5 g						

### 2.2. Identification of the Isolated Compounds

The alkaloids from *Aconitum coreanum* discussed in this paper have been previously identified in a literature report [13]. To confirm the identity of separated compounds, ESI-TOF-MS and ^1^H-NMR were used. Taking compound **1** (GFI) as an example, as shown in Table 3, the ESI-TOF-MS mass spectrum gave the experimental *m/z* 387.0757 and calculated *m/z* 387.0756 as the protonated molecular ion [M] and showed a molecular formula C_23_H_9_N_5_O_2_. The theoretical isotopics of GFI can perfectly match it of actual compound and the mass error under 3 ppm (Table 3). Based on the combined results of TOF-MS and ^1^H-NMR, compound **1** was identified as GFI with its structure given in Figure 1.

**Table 3:** Exact mass measurement and elemental composition of compounds from *Aconitum coreanum*.

Compound	Formula	Selected Ion	Experimental *m/z*	Calcuated *m/z*	Error (mda)	Error (ppm)
GFI (**1**)	C_23_H_9_N_5_O_2_	[M]	387.0757	387.0756	−0.07	−0.19
GFA (**2**)	C_20_H_11_N_7_O_5_	[M]	429.0815	429.0822	0.66	1.54
atisine (**3**)	C_17_H_13_N_9_	[M]	343.1301	343.1294	−0.75	−2.17
GFF (**4**)	C_33_H_15_NO_2_	[M]	457.1088	457.1103	1.53	3.34
GFG (**5**)	C_29_H_9_N_7_O	[M]	471.0861	471.0869	0.81	1.71
GFR (**6**)	C_20_H_11_N_7_O_6_	[M]	485.0995	4850985	−0.98	−2.02
GFP (**7**)	C_37_H_13_N_3_	[M]	499.1127	499.1109	−1.8	−3.6

GFI (**1**): Experimental *m/z* 387.0757 and calculated *m/z* 387.0756 [M], molecular formula C_23_H_9_N_5_O_2_. ^1^H-NMR (600 MHz, CDCl_3_): δ 1.17 (3H, s, 18-H), 2.05 (3H, s, 2-Ac), 4.71 (1H, br. s, 17-H), 4.97 (1H, br. s, 17-H), 5.21 (1H, m, 2-H). Comparing the above data with [14], the obtained product was identified as GFI.

GFA (**2**): Experimental *m/z* 429.0815 and calculated *m/z* 429.0822 [M], molecular formula C_2__0_H_11_N_7_O_5_. ^1^H-NMR (600 MHz, CDCl_3_): δ 1.099 (3H, s, 18-H), 1.977 (3H, s, 13-Ac), 2.065 (3H, s, 2-Ac), 4.779 (1H, br. s, 17-H), 4.92 (1H, br. s, 17-H), 5.04 (1H, d, 13-H), 5.23 (1H, m, 2-H). Comparing the above data with [14], the obtained product was identified as GFA.

atisine (**3**): Experimental *m/z* 343.1301 and calculated *m/z* 343.1301 [M], molecular formula C_17_H_13_N_9_. ^1^H-NMR (600 MHz, CDCl_3_): δ 1.14 (3H, s, 18-H), 4.19 (2H, s, 19-H), 4.42(2H, m, 21-H), 4.74 (2H, m, 22-H), 5.22 (1H, s, 17-H), 5.38 (1H, br. s, 17-H), 8.22(1H, s, 15-OH). Comparing the above data with [15], the obtained product was identified as atisine.

GFF (**4**): Experimental *m/z* 457.1088 and calculated *m/z* 457.1103 [M], molecular formula C_33_H_15_NO_2_. ^1^H-NMR (600 MHz, CDCl_3_): δ 1.132 (3H, s, 18-H), 1.18, 1.20, 2.544 (3H, d,3H, d, 1H, m, 2-isobutyryl), 1.956 (3H, s, 13-Ac), 4.77 (1H, br. s, 17-H), 4.97 (1H, br. s, 17-H), 5.01 (1H, m, 11-H), 5.11 (1H, d, *J* = 18 Hz, 13-H), 5.26 (1H, m, 2-H). Comparing the above data with [16], the obtained product was identified as GFF.

GFG (**5**): Experimental *m/z* 471.0861 and calculated *m/z* 471.0869 [M], molecular formula C_29_H_9_N_7_O. ^1^H-NMR (600 MHz, CDCl_3_): δ 1.122 (3H, s, 18-H), 2.04 (3H, s, 2-Ac), 2.095(3H, s, 11-Ac), 2.182 (3H, s, 13-Ac), 4.858 (1H, s, 17-H), 5.041 (1H, s, 17-H), 5.064 (1H, m, 11-H), 5.12 (1H, d, 13-H), 5.21(1H, m, 2-H). Comparing the above data with [4], the obtained product was identified as GFG.

GFR (**6**): Experimental *m/z* 485.0995 and calculated *m/z* 485.0985 [M], molecular formula C_20_H_11_N_7_O_6_. ^1^H-NMR (600 MHz, CDCl_3_): δ 1.25, 1.27 (3H, s, Me-18, 3'), 1.53, 1.58 (1H, m, H-3, 7), 1.94 (1H, m, H-5), 2.03, 2.04 (3H, s, H-2'', 2'''), 2.15, 2.31 (1H, d, *J* = 18 Hz, H-15), 2.39 (1H, d, *J* = 18 Hz, H-9), 2.47 (2H, q, H-2'), 2.71 (1H, m, H-12), 2.90 (1H, d, *J* = 15 Hz, H-1), 3.16, 3.39 (1H, d, *J* = 12 Hz, H-19), 4.33, 4.85, 5.03 (1H, s, H-20, 17, 17), 5.11 (1H, d, *J* = 9 Hz, H-11), 5.23 (1H, m, H-2). Comparing the above data with [3], the obtained product was identified as GFR.

GFP (**7**): Experimental *m/z* 499.1127 and calculated *m/z* 499.1109 [M], molecular formula C_37_H_13_N_3_. ^1^H-NMR (600 MHz, CDCl_3_): δ 1.11 (3H, s, Me-18), 1.18, 1.22 (3H, d, *J* = 6.6 Hz, *J* = 7.2 Hz, Me-3', 4'), 1.53 (1H, d, *J* = 14.4 Hz, H-3), 1.79 (1H, s, H-3), 2.037, 2.039 (3H, s, H-1''', 2'''), 2.17,(1H, d, *J* = 18 Hz, H-9), 2.28 (1H, m, H-2'), 2.35 (2H, d, *J* = 9 Hz, H-12), 2.71 (1H, m, H-12), 2.90 (1H, d, *J* = 15 Hz, H-1), 3.65, 3.98 (1H, s, H-6, 20), 5.03 (1H, m, H-13), 5.11 (1H, d, *J* = 8.4 Hz, H-11), 5.21 (1H, m, H-2). Comparing the above data with [4], the obtained product was identified as GFP.

## 3. Experimental Section

### 3.1. Apparatus

The pH-zone-refining CCC in the present study is performed with a Model Emilion-300 high-speed counter-current chromatograph (Beijing Emilion Science and Technology Co., Beijing, China) with three PTFE preparative coils connected in series (internal diameter of tube, 1.6 mm; total volume, 300 mL) and a 20 mL sample loop. The β-values of this preparative column ranged from 0.47 at the internal to 0.73 at the external (β = r/R, where r is the rotation radius or the distance from the coil to the holder shaft, and R (R = 7.5 cm) is the revolution radius or the distance between the holder axis and the central axis of the centrifuge). The system was also equipped with a Model NS-1007 constant-flow pump (Beijing Emilion Science and Technology, Beijing, China), a Model 8823A-UV detector (Beijing Emilion Science and Technology, Beijing, China) at 254 nm and a Model 3057 portable recorder (Yokogawa, Sichuan Instrument Factory, Chongqing, China), a model UB-7 pH meter (Denver Instruments, Beijing, China).

The HPLC equipment used was including Waters 600 pump, Waters 600 controller and Waters 996 photodiode array detector (Waters, Milford, MA, USA). Evaluation and quantification were made on an Empower pro data handling system (Waters, Milford, MA, USA).

### 3.2. Reagents and Materials

All solvents used for preparation of crude extracts and pH-zone-refining CCC including petroleum ether (30–60 °C), ethyl acetate, methanol and chloroform were all analytical grades (Damao Chemical Factory, Tianjin, China). The triethylamine (TEA) and hydrochloric acid (HCl) (Laiyang Huagong, Laiyang, China) used for pH-zone-refining CCC were analytical grades. Chromatographic grade methanol (Tedia Company Inc, Fairfield, CT, USA) was used for HPLC analysis. The water used in solutions and dilutions was treated with a Milli-Q water purification system (Millipore, Boston, MA, USA).

The roots of *Aconitum coreanum* (Lèvl) Rapaics were collected from Yunnan province in March 2013. It was after flowering of the plant and the year of vegetation was 2012. It was identified by J. Li (College of Pharmacy, Shandong University of Traditional Chinese Medicine, Jinan, China).

### 3.3. Crude Alkaloids Extracted by Acid-Base Extraction Method

Five kilograms of dried root of *Aconitum coreanum* was ground into a powder and extracted with the heat reflux method for three times with 95% ethanol solution in which 10 mL HCl was added. After filtration, the extracts were combined and evaporated to dryness by rotary vaporization under reduced pressure. Then the residue was dissolved with 2 L of 1% HCl. The acidic extracts were basified to pH 9.5 with NH_3_-water after exacted by petroleum ether. Then it was exacted by chloroform and evaporated to dryness. About 42 g of crude alkaloids obtained were used for further isolation and separation and the yield of crude alkaloids is 0.93%.

### 3.4. Determination of the Partition Coefficient (K)

A small amount of the crude alkaloids is dissolved in a 2 mL volume of the lower phase in a test tube, and analyzed by HPLC to obtain the peak area of the target compound (A_0_). The amount of 2 mL volume of the upper phase was added to the test tube. After the two phases was equilibrated, the lower phase was analyzed to obtain the peak area of the target compound (A_1_). The amount of 5 µL HCl was add to the contents to bring the pH to around 2, and the lower phase was analyzed to obtain the peak area of the target compound (A_2_). Measurement of partition coefficient *K* and *K*_acid_: *K* = (A_0_ − A_1_)/A_1_, *K*_acid_ = (A_0_ − A_2_)/A_2_. If *K* > 1 and *K*_acid_ << 1, the solvent composition is suitable for separation [12].

### 3.5. pH-Zone-Refining CCC Separation Procedure

In this study, pH-zone-refining CCC experiment was performed with a series of two-phase solvent systems composed of Pet–EtOAc–MeOH–H_2_O (5:5:4:6; 5:5:2:8; 5:5:1:9, v/v/v/v) containing 10 mM TEA in upper phase and 10 mM HCl in lower phase. The sample solution was prepared by dissolving 3.5 g crude sample in the mixture of 10 mL upper phase and 10 mL lower phase. CCC separation was performed as follows: the multiplayer coiled column was firstly entirely filled with the upper phase (stationary phase). Then the apparatus was rotated at 850 rpm and the sample solution was injected into the column through sample loop. After that the lower phase (mobile phase) was pumped from the head of the column at a flow rate of 2.0 mL/min. The effluent from the tail end of the column was continuously monitored with a UV detector at 254 nm and the chromatogram was recorded. Each peak fraction was manually collected according to the UV absorbance profile. The pH of each eluted fraction was measured with a pH meter. After the separation was completed, retention of the stationary phase was measured by collecting the column contents into a graduated cylinder by forcing them out of the column with pressurized nitrogen gas. The fractions collected were brought to dryness using a rotary evaporator under reduced pressure and analyzed by HPLC. The separation temperature was at 20 °C.

### 3.6. HPLC Analysis and Identification of CCC Fractions

The crude sample and each peak fraction from CCC were analyzed by HPLC. The analysis was accomplished by a Warters SymmetryShield^TM^ RP18 column (250 mm × 4.6 mm I.D., 5 μm) at room temperature. Acetonitrile and 2 mg/mL sodium 1-heptansulfonate (including 0.2% triethylamine and the pH was adjusted to 3.0 with phosphoric acid) was used as the mobile phase in gradient elution mode as follows: Acetonitrile: 0–10 min, 10 to 30; 10–20 min, 30 to 60; 20–21 min, 60 to 10; 21–30 min, 10. The flow-rate of the mobile phase was 1.0 mL/min. The effluent was monitored at 200 nm by PAD.

### 3.7. TOF-MS and ^1^H-NMR for Identification

The MS instrument used to perform the studies was an electrospray ionization time of flight mass spectrometer Agilent 6520 Q-TOF (Agilent, California, CA, USA), using the operational parameters included in Table 4. The data recorded was processed with the Mass profiler (Agilent, Santa Clara, CA, USA) with accurate mass application specific additions from Agilent MSD TOF software. A second orthogonal sprayer with a reference solution was used as a continuous calibration using the following reference masses: 121.0509 and 922.0098 *m/z*. Spectra were acquired over the *m/z* 50–1000 range at a scan rate of 1 s per spectrum.

**Table 4:** TOP-MS operational parameters.

Parameter	Value
Capillary	3500 V
Nebulizer Pressure	50 psig
Drying Gas	10 L/min
Gas Temperature	350 °C
Fragmentor Voltage	175 V
Skimmer Voltage	60 V
Mass Range	50–1000
Resolution	9500 ± 500 (922.0098)
Reference Masses	121.0509, 922.0098

## 4. Conclusions

The pH-zone-refining CCC method has been developed and successfully applied to the separation and purification of seven diterpenoid alkaloids in the crude extract of *Aconitum coreanum*. Combined HPLC-TOP-MS and ^1^H-NMR analyses were employed to identify the isolated compounds, which were known bioactive compounds. The results indicate that pH-zone-refining CCC has many advantages over conventional CCC, such as an increase of over 10-fold in sample-loading capacity, high purity, and high concentration of the collected fraction and is an efficient method for separation of the kind of alkaloids with low absorbance value.

## References

[1] Bessonova I.A., Yunusov M.S., Kondratèv V.G., Shreter A.I. (1987). Alkaloids of *Aconitum coreanum*. I. Structure of acorine. Chem. Nat. Compd..

[2] Editorial Committee of Chinese Bencao (1998). Chinese Bencao.

[3] Kai J., Yang C.H., Liu J.H., Tang Q.F. (2006). Isolation and identification of Hetisine-type alkaloids from *Aconitum coreanum* by high speed countercurrent chromatography. Acta Pharm. Sin..

[4] Yang C.H., Zhang H.H., Liu J.H. (2004). Alkaloid constituents from root of *Aconitum coreanum*. Chin. Tradit. Herb. Drugs.

[5] Tang Y.Q., Yin Y.M., Huang L., Wang M.H., Yu X.L., Xu J. (2011). Effects of Guanfu total base on experimental atrial fibrillation. Guide Chin. Med..

[6] Wang M.N., Zhu J.N., Yang Y., Li J.D., Huang X.F., Li C.R., Tian Y., Chen X.S. (2008). Effect of acehytisine hydrochloride on vagotonic atrial fibrillation inmongrel dogs. Kokyu To Junkan.

[7] Ito Y. (2005). Golden rules and pitfalls in selecting optimum conditions for high-speed counter-current chromatography. J. Chromatogr. A.

[8] Ito Y. (2013). pH-zone-refining counter-current chromatography: Origin, mechanism, procedure and applications. J. Chromatogr. A.

[9] Zheng Z.J., Wang M.L., Wang D.J., Duan W.J., Wang X., Zheng C.C. (2010). Preparative separation of alkaloids from *Nelumbo nucifera* leaves by conventional and pH-zone-refining counter-current chromatography. J. Chromatogr. B.

[10] Tong S.Q., Yan J.Z., Jian L., Lou J.Z. (2007). Separation of pyridine derivatives from synthetic mixtures by pH-zone-refining counter-current chromatography. J. Sep. Sci..

[11] Fang L., Liu Y.Q., Yang B., Wang X., Huang L.Q. (2011). Separation of alkaloids from herbs using high-speed counter-current chromatography. J. Sep. Sci..

[12] Fang L., Zhou J., Lin Y.L., Wang X., Sun Q.L., Li J.L., Huang L.Q. (2013). Large-scale separation of alkaloids from *Gelsemium elegans* by pH-zone-refining counter-current chromatography with a new solvent system screening method. J. Chromatogr. A.

[13] Tang Q., Yang C., Ye W., Liu J., Zhao S. (2007). Preparative isolation and purification of bioactive constituents from *Aconitum coreanum* by high-speed counter-current chromatography coupled with evaporative light scattering detection. J. Chromatogr. A.

[14] Bessonova I.A. (1999). Alkaloids of *Aconitum coreanum* IX. Tangutisine and 2,11,13-triacetyl-14-hydroxyhetisine. Chem. Nat. Comp..

[15] Razakova D.M., Bessonova I.A., Yunosov M.S. (1988). Atisine chloride and isoatisine from *Aconitum coreanum* and *A. rotundifolium*. Chem. Nat. Comp..

[16] Liu J.H., Hao Z.G., Zhao S.X.J. (1988). Identifica tion of the structure of Guanfu base F. China Pharm. Univ..

